# Blood Neurofilament Light Chain in Parkinson’s Disease: Comparability between Parkinson’s Progression Markers Initiative (PPMI) and Asian Cohorts

**DOI:** 10.3390/jcm10215085

**Published:** 2021-10-29

**Authors:** Jia-Hung Chen, Lung Chan, Chen-Chih Chung, Oluwaseun Adebayo Bamodu, Chien-Tai Hong

**Affiliations:** 1 Department of Neurology, Shuang Ho Hospital, Taipei Medical University, New Taipei City 235, Taiwan; gary.320@hotmail.com (J.-H.C.); 12566@s.tmu.edu.tw (L.C.); 10670@s.tmu.edu.tw (C.-C.C.); 2 Department of Neurology, School of Medicine, College of Medicine, Taipei Medical University, Taipei City 110, Taiwan; 3 Department of Medical Research and Education, Shuang Ho Hospital, Taipei Medical University, New Taipei City 235, Taiwan; 4 Department of Urology, Shuang Ho Hospital, Taipei Medical University, New Taipei City 235, Taiwan; 5 Department of Hematology and Oncology, Cancer Center, Shuang Ho Hospital, Taipei Medical University, New Taipei City 235, Taiwan

**Keywords:** Parkinson disease, neurofilament light chain, NfL, PPMI, diagnosis, biomarker, covariate-adjustment

## Abstract

Elevated blood neurofilament light chain (NfL), which indicates the loss of neuronal integrity, is increasingly implicated as a diagnostic and outcome-predicting biomarker for neurological diseases. However, its diagnostic implication for Parkinson’s disease (PD) remains unclear, with conflicting data reported by several studies. This may result from the demographic heterogeneity of the studied cohorts. The present study investigated the comparability of blood NfL between a domestic, single-centered PD cohort from Shuang Ho Hospital (SHH) in Taiwan, with the large international, multi-center cohort, Parkinson’s Progression Markers Initiative (PPMI). In the SHH PD cohort, with 61 people with PD (PwP) and 25 healthy non-PD controls, plasma NfL unexpectedly was significantly higher in the control group than PwP (14.42 ± 13.84 vs. 9.39 ± 6.91 pg/mL, *p* = 0.05). Interestingly, subgroup analysis revealed a non-significant difference of plasma NfL levels in male PwP compared with controls (8.58 ± 6.21 vs. 7.25 ± 4.43 pg/mL, *p* =0.575), whereas NfL levels were significantly lower in the female PwP group than in their healthy control peers (10.29 ± 7.62 vs. 17.79 ± 15.52 pg/mL, *p* = 0.033). Comparative analysis of the SHH and PPMI cohorts revealed a comparable gender-stratified distribution of blood NfL based on approximate theoretical quantiles. After adjusting for age and gender, no apparent difference in NfL value distribution was observed between the SHH and PPMI cohorts’ control or PD groups. Significant downregulation of blood NfL levels were positively correlated with a reduced probability of having a PD diagnosis in both cohorts. These results demonstrated that the adjustment for demographic background enhances comparability between cohorts, and may be required to eliminate covariate/confounder-associated conflict in blood NfL results between different PD studies. This experience may be beneficial to other researchers around the world who are saddled with limited study participants, especially as data from small cohort sizes are often at greater risk of being skewed by specific variables.

## 1. Introduction

Idiopathic Parkinson disease (PD) is one of the most common neurodegenerative diseases worldwide [1]. It is characterized by rapidly increasing incidence, as evidenced by the 3.6 million newly diagnosed cases globally between 1990 and 2016 [2]. The rise in PD incidence is projected to continue, considering the extended lifespan of both rural and urban populations [1,2]. Despite the diagnostic and therapeutic advances in the last two decades, PD constitutes one of the leading causes of disability. In the United States alone, the economic burden of PD is expected to have increased by an estimated 50% by the year 2037 [3]. 

Currently, PD is diagnosed based on clinical motor symptoms, including bradykinesia, rigidity, resting tremor and postural instability [4]. Yet, neuroimaging remains a vital diagnostic tool. Magnetic resonance imaging (MRI) is vital to exclude secondary parkinsonism, and aids visualization and identification of dorsolateral nigral hyperintensity loss [5,6] and nigral neuromelanin signal change [7,8]. Dopamine transporter single-photon emission computed tomography (DaTscan SPECT) imaging also helps to detect loss of the normal comma/crescent-shaped tracer uptake in the striatum, and is considered beneficial in early diagnosis of PD, especially with the differential diagnosis of PD from a number of non-degenerative parkinsonian disorders with similar clinical features [9,10]. Nonetheless, combining clinical symptoms and neuroimaging still results in substantial misdiagnosis. Besides, neither MRI nor DAT are easily accessible in many regions, and a complementary blood biochemical assessment is warranted for enhancing diagnostic accuracy [11]. Furthermore, the discovery of veritable biomarkers may facilitate the accurate prediction of a long-term clinical outcome, and the parameter of the treatment response in clinical trial.

In recent decades, several biomarkers in the body fluid, such as orexin, 8-Hydroxy-2′-Deoxyguanosine, peripheral proteasomes, caspases, dopamine, dopamine receptor, alpha-synuclein, and apolipoprotein A1 (ApoA1), have been suggested to be diagnostic of PD [11]; however, some of these biomarkers fail to reflect the disease course while others only exhibit vague results. Recently, significant aberration in neurofilament light chain (NfL) levels have been associated with Alzheimer’s disease (AD) [12], amyotrophic lateral sclerosis [13], multiple sclerosis [14] and cerebral vascular disease [15]. NfL, a 68 kDa neuronal cytoplasmic protein, is considered critical for radial growth and axon stability, and the release of NfL into blood and/or cerebrospinal fluid (CSF) circulation reflects axonal damage [16,17]. There is further evidence to suggest that altered NfL levels are correlated with the disease severity and progression in both motor and cognitive functions of patients with PD [18]. A recently completed 5-year study by Brit Mollenhauer et al, under the auspices of the Parkinson’s Progression Markers Initiative (PPMI), reported elevated Nfl levels in the blood of patients with PD or high risk of PD compared to healthy control subjects, and that these NfL levels increased over time and positively correlated with exacerbated motor symptoms [19]. 

However, contrary to the findings of this seminal report, several studies have reported inconsistencies or no significant differences in the blood NfL levels between people with PD (PwP) and healthy controls [20,21,22,23]. The discrepancy raised concerns regarding the application blood NfL as a diagnostic and predicting biomarker for PD. The discrepancy may be associated with the difference in the baseline demographic factors, including the age, sex and disease duration of each PD cohort. In order to circumvent the probable cause of reported discrepancy, we made a direct cohort–cohort comparison after adjusting for these confounding factors. Thus, the present study comparatively evaluated the blood NfL from a small-scale, single-centered PD cohort with the large-scale, multinational PPMI study to delineate the possible origin of the inconsistency in the altered blood NfL in PD.

## 2. Results

### 2.1. The SHH Cohort

Plasma NfL values were evaluated in the SHH cohort (*n* = 86). Sixty-one (70.9%) were patients with PD (PwP), while 29.1% were non-PD healthy control volunteers. Demographic data of all participants are shown in Table 1. The PwP group consisted of 29 males and 32 females. The control group, on the other hand, had 17 males and 8 females. While we found no statistically significant difference in the ages (66.77 ± 6.38 vs. 68.04 ± 8.97 years old, *p* = 0.56), gender (female 32 vs. 8, *p* = 0.10), MMSE scores (26.64 ± 2.99 vs. 27.00 ± 3.55, *p* = 0.28), and MoCA scale (22.28 ± 4.84 vs. 22.72 ± 4.10, *p* = 0.88) of the PwP and control groups, we observed significantly higher plasma NfL values in the control group than in the PwP group (14.42 ± 13.84 vs. 9.39 ± 6.91 pg/mL, *p* = 0.05). Interestingly, subgroup analysis revealed non-significantly higher NfL value in the male PwP group than in the controls (8.58 ± 6.21 vs. 7.25 ± 4.43 pg/mL, *p* = 0.58), but lower NfL levels in the female PwP group than in their healthy control peers (10.29 ± 7.62 vs. 17.79 ± 15.52 pg/mL, *p* = 0.03).

### 2.2. The PPMI Cohort

In the PPMI cohort, there was a total of 3156 eligible cases (male = 2052, female = 1104) with available serum NfL data, which were allocated to either the PD, control, or prodromal group, according to their clinical diagnoses. In PD group, there were 1248 males with mean NfL value 15.2 ± 11.2 pg/mL and 681 females with mean NfL value 15.8 ± 11.8 pg/mL. In the control group, there were 584 males with mean NfL value 12.4 ± 6.52 and 360 females with mean NfL value 12.7 ± 7.48. In the prodromal group, there were 220 males with mean NfL value 17.2 ± 9.23 and 63 females with mean NfL value 21.3 ± 24.8. The PD and prodromal groups exhibited higher serum NfL values, compared to the controls, regardless of gender.

### 2.3. Comparison between the SHH and PPMI Cohort

**Figure 1:**
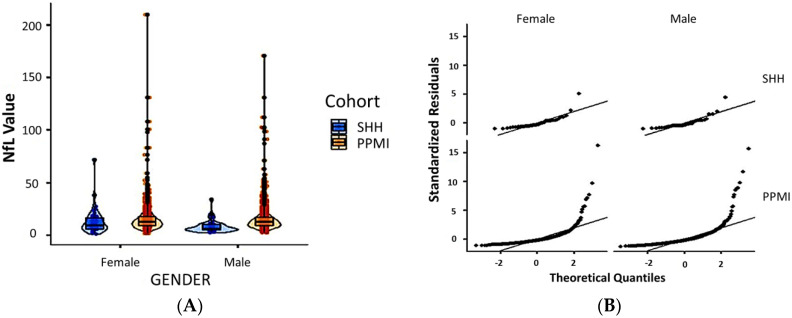
(**A**) Violin plot with imbedded boxplots showing the gender-stratified blood NfL value in the SHH and PPMI cohorts. (**B**) Graphical representation of the standardized residuals vs. theoretical quantiles of blood NfL values in females or males from the SHH and PPMI cohorts. SHH, Shuang Ho Hospital; PPMI, Parkinson’s Progression Markers Initiative.

**Figure 2:**
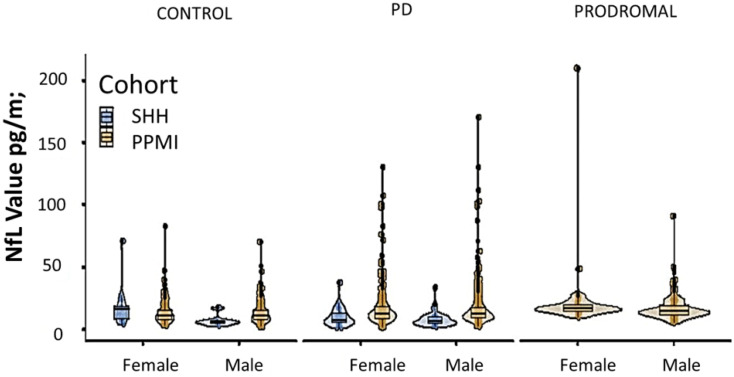
Violin plot with imbedded boxplots showing the blood NfL levels in control, PD, or prodromal cases from the SHH and PPMI cohorts, stratified based on gender. SHH, Shuang Ho Hospital; PPMI, Parkinson’s Progression Markers Initiative.

**Figure 3:**
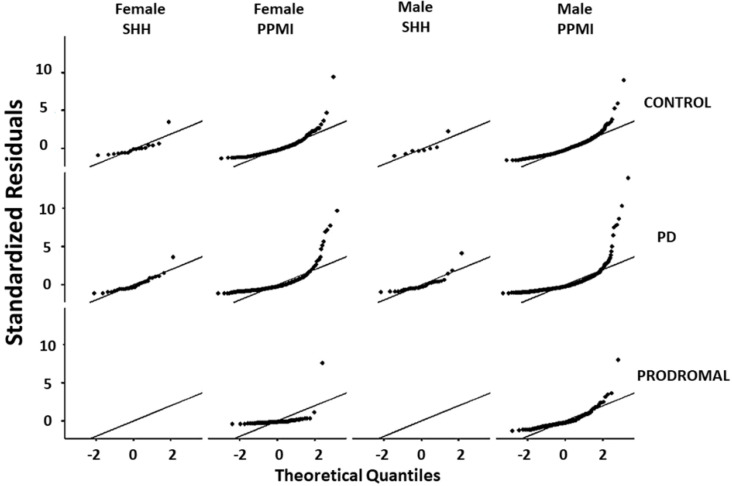
Graphical representation of the standardized residuals vs. theoretical quantiles of blood NfL values in control, PD, or prodromal cases from male or female participants in the SHH and PPMI cohorts. SHH, Shuang Ho Hospital; PPMI, Parkinson’s Progression Markers Initiative.

**Figure 4:**
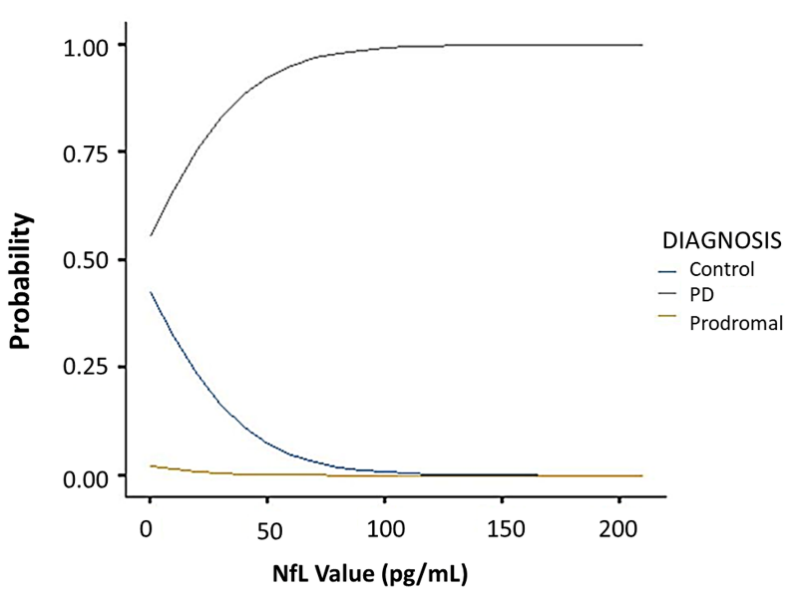
Multinominal logistic regression curves showing the correlation between blood NfL value and the probability of being classed into the control, PD, or prodromal group in pooled PPMI and SHH cohorts. SHH, Shuang Ho Hospital; PPMI, Parkinson’s Progression Markers Initiative.

Comparative analysis of the SHH and PPMI cohorts showed a comparable, gender-stratified distribution of blood NfL based on approximate theoretical quantiles, regardless of PD or non-PD diagnosis (Figure 1A,B). In both the SHH and PPMI cohorts, the blood NfL values were higher in patients with PD, except for female healthy control volunteers from the SHH cohort, who exhibited higher NfL levels than their PD counterparts (Figure 2). However, after adjusting for age and gender, no apparent difference in NfL value distribution was observed between the SHH and PPMI cohorts’ control or PD groups (Figure 3). Consistent with these observations, results from multinominal logistic regression of pooled SHH and PPMI data showed a steep rise in the probability of PD diagnosis as blood NfL value increases to ~100 pg/mL before plateauing. Conversely, a significant downregulation of blood NfL levels was positively correlated with a reduced probability of having a PD diagnosis (Figure 4).

## 3. Discussion

The present study demonstrated the comparability of blood NfL values between a single-centered Asian cohort (SHH) and the large-scale PD cohort from multiple western countries (PPMI). Interestingly, despite the higher plasma NfL in the SHH-healthy controls, especially in the female, when compared with the PwP, this inter-cohort difference was eliminated when adjusted for selected demographic factors. This finding suggests the potential role of cohort size and/or certain demographic variables, including the classic covariates age and gender, in the observed discrepancy between cohorts. 

In the last two decades, blood NfL has been touted as a potentially reliable diagnostic biomarker of PD, based on its constitutive ability to reflect neuronal injury. NfL is a neuron-specific protein which presents as a main component of axons and also a byproduct of nerve cell degeneration. Increasing NfL levels in the blood and CSF reflects the degree of axonal damage in various neurological disorders. In neurodegenerative diseases, the progressive loss of neurons in central nervous system causes the damage to myelinated axons, leading to NfL release to the blood and CSF. PD, akin to other neurodegenerative diseases, is expected to exhibit an elevated pattern of blood NfL in PwP compared with controls. However, studies from different PD cohorts globally have failed to demonstrate a universal result to validate this hypothesis. This dearth of validation has limited the further application of blood NfL as a biomarker to improve the diagnostic accuracy of PD. Additionally, a reliable biomarker is also essential for assessing treatment response in the clinical trial for disease modification. A meta-analysis may be helpful to pool all the results from different cohorts to conclude the finding; however, the detailed demographic data of each case in all the cohorts usually remain unknown. Thanks to the open-database policy of PPMI, the present study conducts another approach and makes a detailed comparison between a relatively small single-center cohort with the large multi-center PPMI cohort to identify the effect of differences in demographic factors on the blood NfL in PwP. 

Our findings corroborate the results from the PPMI, which indicates the elevation of serum NfL levels in PwP, and the upregulation of NfL positively correlates with age, duration of morbidity, and disease severity [19]. By attributing the observed difference in the NfL profiles of female and male patients with PD from the SHH and PPMI cohorts to sample size- and age-distribution-associated biases, our comparative analyses of data from the two cohorts highlight the role of factor adjustment in bias minimization. Indeed, factor adjustment amplified the similarity in the NfL value distribution observed in both our SHH cohort and the PPMI cohort regardless of age-, gender-, or diagnosis-stratification. Moreover, akin to the PPMI report [19], the probability of PD diagnosis also increased with elevated blood NfL value in our SHH cohort. Our subsequent results showing that the plasma NfL value is enhanced in both male and female Taiwanese PwP lends credence to the position that, as with most studies with a relatively small cohort size, certain results are modifiable upon the expansion of cohort size. For context, the observed higher blood NfL in female control volunteers compared to the PwP group initially raised some concerns about the clinical application of blood NfL in female Taiwanese PwP. However, this concern was alleviated after obtaining an identical NfL distribution pattern between SHH and PPMI cohorts after statistical adjustment. This finding is also indicative, at least in part, of the consistency of blood NfL profile across countries and cohorts. This experience may be beneficial to other researchers around the world who are saddled with a limited study number of participants, especially as data from such small cohort size are often at greater risk of being skewed by specific variables, and thus increasing the odds of data inconsistency with contemporary studies or outcome misinterpretation. 

Other studies have also suggested a supplementary role for altered NfL levels, either in plasma or cerebrospinal fluid, to the known PD-pathognomonic biomarker α-synuclein for differentiating patients with PD patients from non-PD controls [20,21]. Moreover, some studies have also reported an inverse correlation between NfL level and cognitive function, with higher serum NfL being associated with poor cognition in PwP [22,23,24]. In fact, several reports have highlighted the association between altered NfL levels and the severity of motor function impairment and/or worsening cognition in patients with PD [25,26]. Certain questions remain regarding the adoption of NfL as a biomarker of diagnosis in PD [27], such as: what is the probable explanation for documented elevated CSF NfL value only in patients with PD spanning more than 2 years? [28]. Furthermore, another study found that the temporal dynamics of serum NfL levels in individuals were associated with the conversion from prodromal to PD, suggesting that NfL may not be an early, PD-specific diagnostic biomarker [29]. These remaining questions necessitate sophisticated global data-pooling with consideration of background demographic data to fully assess the role of blood NfL as a reliable diagnostic biomarker in persons at high risk of PD.

The present study has some limitations. First, plasma NfL was measured in our SHH cohort, instead of the serum NfL measured in the PPMI database. However, the equivalence of serum and plasma NfL has been reported in a recent published work [30]. Second, the statistical comparison of probability distribution between the SHH and PPMI cohorts was based on quantiles and not absolute NfL values. Third, unlike the large multicenter PPMI cohort, our SHH cohort size was relatively small and did not include a prodromal group. Fourth, we did not analyze the effect of disease duration, which might also influence the value of plasma NfL.

## 4. Materials and Methods

### 4.1. Study Participants

The present observatory, single-center, longitudinal cohort study enrolled a total of 86 participants, consisting of 61 patients with PD and 25 healthy non-PD controls, who presented at Shuang Ho Hospital between November 2017 and September 2019. The diagnosis of PD was based on the UK Parkinson’s Disease Society Brain Bank Diagnostic Criteria [31]. Only subjects with mild to moderate PD, defined as Stage I to III according to the modified Hoehn and Yahr scale, were included in the study. For the control group, enrolled participants were free from any known dementia, as defined by a mini-mental state examination (MMSE) score <26, psychiatric symptoms, malignancy, major systemic disorders such as uncontrolled hypertension, poor controlled diabetic mellitus, unstable coronary artery disease, chronic kidney disease, or any established neurodegenerative disease. The healthy control volunteers were regularly followed-up in outpatient clinics for any neurological conditions including transient ischemic attack, dizziness, headache, and diabetic polyneuropathy.

For all participants, patho-epidemiological data such as age, sex, morbidity status, disease duration, and cognitive information were collected during enrollment. Disease severity for all participants with PD was determined using the Unified Parkinson’s Disease Rating Scale (UPDRS) Parts I, II, and III. Blood samples were obtained from both PD and control groups for assessment of plasma NfL. This study was approved by the Joint Institutional Review Board of Taipei Medical University (Approval No. N201609017 and N201801043).

#### 4.1.1. Neurofilament Light Chain Immunomagnetic Reduction Assay

The quantification of plasma NfL was assessed using immunomagnetic reduction (IMR) technology, strictly following earlier described protocol [32]. The IMR reagent consisted of antibody-functionalized Fe_3_O_4_ magnetic nanoparticles in cold 1X phosphate-buffered saline (PBS) solution at a 1:1 ratio. The mean diameter of the magnetic nanoparticles was 53 nm, as measured by laser dynamic scattering. The concentration of the NfL reagent was 10 mg Fe/mL. The reagent was stored at 4 °C. Anti-NF-L antibody (8A1) (Cat. # sc-20012, Santa Cruz Biotechnology, Inc., Santa Cruz, CA, USA) was used to determine probable association between magnetic nanoparticles and NfL. Specific association between magnetic nanoparticles and NfL was confirmed when the anti-NF-L antibody (Cat. # sc-20012, Santa Cruz) covalently bonded to dextran, which acted as an interface between the antibody and the Fe_3_O_4_ core of the nanoparticles.

#### 4.1.2. The PPMI Cohort

The Parkinson’s Progression Markers Initiative (PPMI) was founded by the Michael J. Fox Foundation for PD research development, specifically to carry out large-cohort observational studies aiming to define and measure reliable biomarkers for predicting disease progression in patients with PD, and to create a pipeline for the accelerated development and clinical application of novel disease-modifying therapeutics. All clinical information, image, and biologic data used in this present study were accessed and retrieved after proper authorization was obtained by our team. The retrieved PPMI cohort data, including NfL value and demographic information, were screened for eligibility. Finally, 3156 eligible cases were enrolled for further analysis, including 1929 PD cases, 283 prodromal cases, and 944 healthy controls.

### 4.2. Covariate-Adjusted Regression 

Confounding adjustment was carried out as previously described [33,34]. To adjust for confounders, we performed covariate-adjusted regression, wherein our outcome variable, namely, diagnosis of PD, was regressed on the exposure variables and covariates. Since the results’ validity relies on the correct specification of our regression model, we included all the covariates, interactions, and functions with probable influence on the exposure–outcome association. Upon meeting these conditions, the parameter associated with altered NfL levels determined the difference in the PD diagnosis by including NfL in any set of fixed values of other covariates

### 4.3. Statistical Analysis

All statistical analyses were performed using IBM SPSS Statistics for Windows 10 (IBM Corp. Released 2010. IBM SPSS Statistics for Windows, Version 19.0. Armonk, NY, USA: IBM Corp.) and JMP® software, Version 13 (SAS Institute Inc., Cary, NC, USA, 1989–2019). Chi-square (*X*^2^) was used to compare the gender distribution between patients with PD and healthy controls. Independent Student’s *t*-test was used to determine the difference in age, Mini-Mental State Examination (MMSE), Montreal Cognitive Assessment (MoCA), and NfL values between two groups. By delineating confounders (variables related to both the variability in serum NfL and the diagnosis of PD but lack causal implication) from covariates (variables that explain aspects of variability in PD diagnosis but are not influenced by variation in serum NfL), we adjusted for classic covariates, namely age and sex. The association between serum NfL level and diagnosis of PD in all study participants was determined using a multivariable logistic regression, with adjustment for age and sex. *p*-Value < 0.05 was considered statistically significant.

Multinomial logistic regression was performed to identify the independent variables that remained associated with the PD diagnosis, such as age, sex, morbidity status, disease duration, and cognitive information including Unified Parkinson’s Disease Rating Scale (UPDRS) Parts I, II, III, and plasma NfL level. This model used the Pearson’s χ2 statistical test, and the data obtained were analyzed using IBM SPSS Statistics for Windows 10 (IBM Corp. Released 2010. IBM SPSS Statistics for Windows, Version 19.0. Armonk, NY, USA: IBM Corp.) and JMP® software, Version 13 (SAS Institute Inc., Cary, NC, USA,1989–2019).

## 5. Conclusions

Our results demonstrated that upregulated plasma NfL value is a diagnostic biomarker of PD; however, this biomarker is susceptible to small-cohort-size-introduced bias. We also provided some evidence that adjustment for demographic background enhances the comparability of our small local cohort with a large-size, international cohort. The inter-cohort statistical comparison of probability distribution based on quantiles may present a method to overcome possible bias and avoid inconsistency between studies.

## Figures and Tables

**Table 1:** Demographic data of the SHH cohort.

	PwP	Control	*p*-Value
Number of patients (*n*)	61	25	
Age (years, mean ± SD)	66.77 ± 6.38	68.04 ± 8.97	0.56
Male (*n*)	29	17	
Female (*n*)	32	8	0.10
Disease duration (years, mean ± SD)	2.61 ± 2.22	N/A	
MMSE (mean ± SD)	26.64 ± 2.99	27.00 ± 3.55	0.28
MoCA (mean ± SD)	22.28 ± 4.84	22.72 ± 4.10	0.88
UPDRS-I (mean ± SD)	2.20 ± 1.67	N/A	
UPDRS-II	7.61 ± 5.63	N/A	
UPDRS-III	21.84 ± 8.65	N/A	
Plasma NfL (all, mean ± SD)	9.39 ± 6.91	14.42 ± 13.84	0.05 *
Male	8.58 ± 6.21	7.25 ± 4.43	
Female	10.29 ± 7.62	17.79 ± 15.52	

MMSE, Mini-Mental State Examination; MoCA, Montreal Cognitive Assessment; UPDRS, Unified Parkinson’s Disease Rating Scale; *, *p* < 0.05.

## Data Availability

Publicly available datasets were analyzed in this study. This data can be found here: https://www.ppmi-info.org/access-data-specimens/download-data/ (assessed on 15 October 2019).

## References

[1] Erkkinen M.G., Kim M.O., Geschwind M.D. (2018). Clinical Neurology and Epidemiology of the Major Neurodegenerative Diseases. Cold Spring Harb. Perspect. Biol..

[2] Dorsey E., Elbaz A., Nichols E., Abd-Allah F., Abdelalim A., Adsuar J., Ansha M.G., Brayne C., Choi J.-Y.J., Collado-Mateo D. (2018). Global, regional, and national burden of Parkinson’s disease, 1990–2016: A systematic analysis for the Global Burden of Disease Study 2016. Lancet Neurol..

[3] Yang W., Hamilton J.L., Kopil C., Beck J.C., Tanner C.M., Albin R.L., Ray Dorsey E., Dahodwala N., Cintina I., Hogan P. (2020). Current and projected future economic burden of Parkinson’s disease in the U.S. NPJ Parkinsons Dis..

[4] Poewe W., Seppi K., Tanner C.M., Halliday G.M., Brundin P., Volkmann J., Schrag A.E., Lang A.E. (2017). Parkinson disease. Nat. Rev. Dis. Primers.

[5] Schwarz S.T., Afzal M., Morgan P.S., Bajaj N., Gowland P.A., Auer D.P. (2014). The ‘swallow tail’ appearance of the healthy nigrosome—a new accurate test of Parkinson’s disease: A case-control and retrospective cross-sectional MRI study at 3T. PLoS ONE.

[6] Bae Y.J., Kim J.M., Kim E., Lee K.M., Kang S.Y., Park H.S., Kim K.J., Kim Y.E., Oh E.S., Yun J.Y. (2016). Loss of nigral hyperintensity on 3 Tesla MRI of Parkinsonism: Comparison with (123) I-FP-CIT SPECT. Mov. Disord..

[7] Castellanos G., Fernandez-Seara M.A., Lorenzo-Betancor O., OrtegaCubero S., Puigvert M., Uranga J., Vidorreta M., Irigoyen J., Lorenzo E., Muñoz-Barrutia A. (2015). Automated neuromelanin imaging as a diagnostic biomarker for Parkinson’s disease. Mov. Disord..

[8] Langley J., Huddleston D.E., Sedlacik J., Boelmans K., Hu X.P. (2016). Parkinson’s disease-related increase of T2*-weighted hypointensity in substantia nigra pars compacta. Mov. Disord..

[9] Ba F., Martin W.R. (2015). Dopamine transporter imaging as a diagnostic tool for parkinsonism and related disorders in clinical practice. Parkinsonism Relat. Disord..

[10] Gayed I., Joseph U., Fanous M., Wan D., Schiess M., Ondo W., Won K.S. (2015). The impact of DaTscan in the diagnosis of Parkinson disease. Clin. Nucl. Med..

[11] Emamzadeh F.N., Surguchov A. (2018). Parkinson’s Disease: Biomarkers, Treatment, and Risk Factors. Front Neurosci..

[12] Forgrave L.M., Ma M., Best J.R., DeMarco M.L. (2019). The diagnostic performance of neurofilament light chain in CSF and blood for Alzheimer’s disease, frontotemporal dementia, and amyotrophic lateral sclerosis: A systematic review and meta-analysis. Alzheimers Dement Amst..

[13] Sun Q., Zhao X., Li S., Yang F., Wang H., Cui F., Huang X. (2020). CSF Neurofilament Light Chain Elevation Predicts ALS Severity and Progression. Front Neurol..

[14] Kapoor R., Smith K.E., Allegretta M., Arnold D.L., Carroll W., Comabella M., Furlan R., Harp C., Kuhle J., Leppert D. (2020). Serum neurofilament light as a biomarker in progressive multiple sclerosis. Neurology.

[15] Nielsen H.H., Soares C.B., Høgedal S.S., Madsen J.S., Hansen R.B., Christensen A.A., Madsen C., Clausen B.H., Frich L.H., Degn M. (2020). Acute Neurofilament Light Chain Plasma Levels Correlate With Stroke Severity and Clinical Outcome in Ischemic Stroke Patients. Front. Neurol..

[16] Barro C., Chitnis T., Weiner H.L. (2020). Blood neurofilament light: A critical review of its application to neurologic disease. Ann. Clin. Transl. Neurol..

[17] Bridel C., van Wieringen W.N., Zetterberg H., Tijms B.M., Teunissen C.E., Alvarez-Cermeño J.C., Andreasson U., Axelsson M., Bäckström D.C., The NFL Group (2019). Diagnostic Value of Cerebrospinal Fluid Neurofilament Light Protein in Neurology: A Systematic Review and Meta-analysis. JAMA Neurol..

[18] Lin C.H., Li C.H., Yang K.C., Lin F.J., Wu C.C., Chieh J.J., Chiu M.J. (2019). Blood NfL: A biomarker for disease severity and progression in Parkinson disease. Neurology.

[19] Mollenhauer B., Dakna M., Kruse N., Galasko D., Foroud T., Zetterberg H., Schade S., Gera R.G., Wang W., Gao F. (2020). Validation of Serum Neurofilament Light Chain as a Biomarker of Parkinson’s Disease Progression. Mov. Disord..

[20] Oosterveld L.P., Verberk I.M.W., Majbour N.K., El-Agnaf O.M., Weinstein H.C., Berendse H.W., Teunissen C.E., van de Berg W.D.J. (2020). CSF or serum neurofilament light added to α-Synuclein panel discriminates Parkinson’s from controls. Mov. Disord..

[21] Hansson O., Janelidze S., Hall S., Magdalinou N., Lees A.J., Andreasson U., Norgren N., Linder J., Forsgren L., Constantinescu R. (2017). Swedish BioFINDER study. Blood-based NfL: A biomarker for differential diagnosis of parkinsonian disorder. Neurology.

[22] Sampedro F., Pérez-González R., Martínez-Horta S., Marín-Lahoz J., Pagonabarraga J., Kulisevsky J. (2020). Serum neurofilament light chain levels reflect cortical neurodegeneration in de novo Parkinson’s disease. Parkinsonism Relat. Disord..

[23] Marques T.M., van Rumund A., Oeckl P., Kuiperij H.B., Esselink R.A.J., Bloem B.R., Otto M., Verbeek M.M. (2019). Serum NFL discriminates Parkinson disease from atypical parkinsonisms. Neurology.

[24] Lin Y.S., Lee W.J., Wang S.J., Fuh J.L. (2018). Levels of plasma neurofilament light chain and cognitive function in patients with Alzheimer or Parkinson disease. Sci. Rep..

[25] Ng A.S.L., Tan Y.J., Yong A.C.W., Saffari S.E., Lu Z., Ng E.Y., Ng S.Y.E., Chia N.S.Y., Choi X., Heng D. (2020). Utility of plasma Neurofilament light as a diagnostic and prognostic biomarker of the postural instability gait disorder motor subtype in early Parkinson’s disease. Mol. Neurodegener..

[26] Chen C.H., Lee B.C., Lin C.H. (2020). Integrated Plasma and Neuroimaging Biomarkers Associated with Motor and Cognition Severity in Parkinson’s Disease. J. Parkinsons Dis..

[27] Zetterberg H. (2020). Is there a value of Neurofilament Light as a biomarker for neurodegeneration in Parkinson’s Disease?. Mov. Disord..

[28] Lerche S., Wurster I., Röben B., Zimmermann M., Machetanz G., Wiethoff S., Dehnert M., Rietschel L., Riebenbauer B., Deuschle C. (2020). CSF NFL in a Longitudinally Assessed PD Cohort: Age Effects and Cognitive Trajectories. Mov. Disord..

[29] Wilke C., Dos Santos M.C.T., Schulte C., Deuschle C., Scheller D., Verbelen M., Brockmann K., Von Thaler A., Sünkel U., Roeben B. (2020). Intra-individual Neurofilament Dynamics in Serum Mark the Conversion to Sporadic Parkinson’s Disease. Mov. Disord..

[30] Qiu X., Lee S., Jackson J., Zhao X., Shields A., Matias M., Uzgiris A. (2020). Equivalence of serum and plasma neurofilament light chain levels using highly sensitive automated immunoassay. Alzheimers Dement..

[31] Clarke C.E., Patel S., Ives N., Rick C.E., Woolley R., Wheatley K., Walker M.F., Zhu S., Kandiyali R., Yao G. (2016). Clinical effectiveness and cost-effectiveness of physiotherapy and occupational therapy versus no therapy in mild to moderate Parkinson’s disease: A large pragmatic randomised controlled trial (PD REHAB). Health Technol. Assess..

[32] Liu H.C., Lin W.C., Chiu M.J., Lu C.H., Lin C.Y., Yang S.Y. (2020). Development of an assay of plasma neurofilament light chain utilizing immunomagnetic reduction technology. PLoS ONE.

[33] Li L., Kleinman K., Gillman M.W. (2014). A comparison of confounding adjustment methods with an application to early life determinants of childhood obesity. J. Dev. Orig. Health Dis..

[34] Groenwold R.H.H., Palmer T.M., Tilling K. (2021). To Adjust or Not to Adjust? When a “Confounder” Is Only Measured After Exposure. Epidemiology.

